# Hypokalemic Periodic Paralysis in a Young Woman With Mast Cell Activation Syndrome: A Case Report of an Atypical Presentation Associated With an Ultra‐Rare CACNA1S Variant

**DOI:** 10.1002/ccr3.72808

**Published:** 2026-05-29

**Authors:** Ali Moradi, Yasmin Aboutaleb, Saba Noreen, Abdullah Sahyouni, Hasin Sharma, Olugbenga Oyesanmi, Ian M. Kahane, Yizhi Lin, Hakan R. Toka

**Affiliations:** ^1^ Internal Medicine HCA Florida Blake Hospital, University of South Florida (USF), Morsani College of Medicine Bradenton USA; ^2^ Nova Southeastern University, Kiran C. Patel College of Osteopathic Medicine Florida USA; ^3^ Manatee Kidney Diseases Consultants Bradenton USA

**Keywords:** CACNA1S mutation, episodic paralysis, hKPP, hypokalemia, hypokalemic periodic paralysis, mast cell activation syndrome, MCAS

## Abstract

Hypokalemic periodic paralysis (hKPP) is a rare neuromuscular channelopathy characterized by transient episodes of muscle weakness or paralysis associated with low serum potassium levels. It has been most commonly linked to autosomal dominant mutations in ion channel genes, specifically CACNA1S and SCN4A, which impair skeletal muscle excitability. Although hKPP typically presents in adolescent males with a positive family history, atypical cases in women may be underrecognized due to milder or less frequent attacks and non‐specific symptoms between episodes. We present the case of a 31‐year‐old female with a history of confirmed mast cell activation syndrome (MCAS) and no family history of similar symptoms, who presented with episodes of weakness, muscle cramps, and presyncope, typically triggered by high‐carbohydrate meals or exercise. During one of these attacks, she was found to have severe hypokalemia. Although mast cell activation syndrome can also cause weakness and hypokalemia with certain triggers, this patient did not experience flushing or urticaria during the episodes. Given her atypical presentation, including weakness rather than flaccid paralysis commonly seen in hKPP, and the absence of typical symptoms of MCAS, genetic testing was performed. This revealed an ultra‐rare heterozygous variant in the CACNA1S gene (NM_000069.3: c.3844G > T; p.A1282S), classified as a variant of uncertain significance (VUS). When aligning protein sequences, Alanine at amino acid position 1282 is evolutionary conserved among species (human, mouse, bovine, whale); however, no functional studies on A1282S exist. In conclusion, maintaining a high index of suspicion for hKPP in patients presenting with nonspecific fatigue or weakness associated with hypokalemia and identifiable triggers is important. Although genetic testing is not always required, in cases with autosomal dominant inheritance, it can be useful in patients without family history, nonspecific symptoms or when the differential diagnosis remains challenging.

## Key Clinical Message

In patients presenting with fatigue, presyncope, or weakness associated with hypokalemia, hypokalemic periodic paralysis should be considered. If the patient has other conditions, such as mast cell activation syndrome, which can cause similar symptoms, genetic testing may be useful after ruling out secondary causes, including hyperthyroidism.

## 1 Introduction

Hypokalemic periodic paralysis (hKPP) is a rare, episodic neuromuscular disorder characterized by transient, often flaccid, muscle weakness or paralysis occurring in the setting of low serum potassium levels. hKPP results from dysfunction of ion channels essential for maintaining muscle membrane excitability. The pathophysiology is most commonly associated with autosomal dominant mutations in the CACNA1S gene, which encodes the alpha‐1 subunit of a voltage‐dependent L‐type calcium channel, or in the SCN4A gene, which encodes a subunit of the skeletal muscle sodium channel [1, 2, 3, 4, 5]. These mutations cause abnormal depolarization of skeletal muscle fibers during hypokalemia. The estimated prevalence of hKPP is approximately 1 in 100,000 individuals, with a marked male predominance and a male‐to‐female ratio ranging from 3:1 to 4:1 [2]. The gender difference can be explained by penetrance, which is higher in males likely due to sex hormone effects that modulate ion channel activity and disease expression. The condition typically presents during the first or second decade of life, often between ages 10 and 35 [5]. The classic presentation includes episodes of generalized muscle weakness or paralysis that often involve the proximal muscles more than the distal muscles, especially in the lower extremities [4]. The triggers for hKPP episodes include carbohydrate‐rich meals, rest after vigorous exercise, emotional stress, alcohol consumption, and medications such as insulin, beta‐agonists, or corticosteroids [2, 3, 4]. In between episodes, most patients are clinically normal, although some may develop fixed muscle weakness over time due to repeated muscle damage [6]. While hKPP is often considered a genetic disease, acquired forms exist, most notably thyrotoxic periodic paralysis [7, 8]. Diagnosing hKPP is often challenging, especially in patients who do not fit the classic profile. Furthermore, serum potassium levels may normalize between episodes, making it easy to overlook unless labs are drawn during an attack [3]. Evaluation should include a comprehensive metabolic panel during and between attacks, thyroid function tests, electrocardiogram (ECG), and, if possible, electromyography (EMG) during an attack. Although genetic testing for CACNA1S or SCN4A mutations can confirm the diagnosis, it is not always necessary and may not be readily available or affordable [5]. In clinical practice, diagnosis is often based on history, clinical pattern, exclusion of alternative causes, and the therapeutic response to potassium replacement [4]. A helpful confirmatory tool is the long exercise test, a form of repetitive nerve stimulation test, which has shown a sensitivity of up to 95% in patients with frequent attacks [9]. Treatment of hKPP consists of acute and preventative management. During an attack, oral or intravenous potassium chloride is administered depending on severity and risk of arrhythmia. Potassium must be corrected cautiously to avoid rebound hyperkalemia [1, 2, 3]. For long‐term management, patients are advised to avoid known triggers, and pharmacologic prevention has been shown to reduce attack frequency [1, 6]. In patients with contraindications or cost concerns, potassium‐sparing diuretics such as spironolactone or eplerenone may also be used as maintenance therapy [3].

## 2 Case History

**FIGURE 1:**
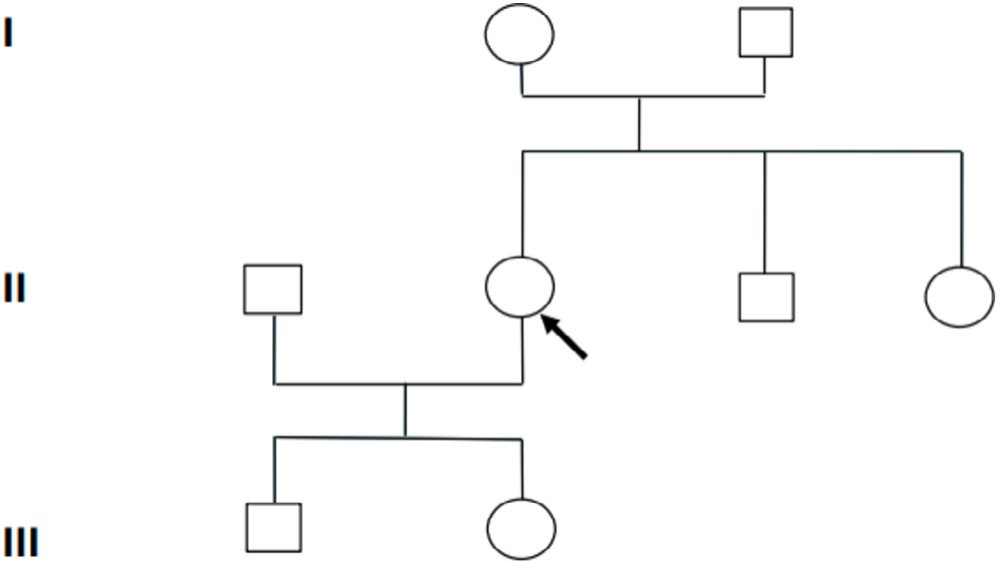
Three‐generation pedigree of the proband (arrow) with hypokalemic periodic paralysis and CACNA1S p.Ala1282Ser variant. No family members are known to have similar episodic weakness or documented hypokalemia. Formal segregation analysis could not be performed because parental samples were not available (circles = females, squares = males).

A 31‐year‐old Hispanic female patient with a medical history of panic disorder and mast cell activation syndrome (MCAS) presented to Clinic for evaluation of recurrent presyncope, dizziness, muscle cramps, and fatigue for which she went to the Emergency Department (ED). During ED visit, she was found to have hypokalemia with a serum potassium of 2.0 mEq/L (reference range: 3.5–5.0 mEq/L). At that time, her evaluation, including vital signs, brain MRI, EEG, and laboratory studies, was otherwise unremarkable. Over the past few months, she reported multiple episodes of presyncope and muscle cramps in the lower extremities, accompanied by dizziness, lightheadedness, and nausea, most frequently during physical activity. One episode of dizziness and fatigue occurred while driving, leading her to discontinue driving and rely on public transportation. She also endorsed unintentional weight loss of approximately 7 kg and recurrent cramps involving her legs and neck. She had been followed by an allergist for the past 2 years for MCAS, during which she occasionally experienced elevations in blood pressure and heart rate in response to certain foods. Notably, she recalled being told of low potassium during both of her prior pregnancies. She denied any family history of similar symptoms or related conditions. A three‐generation pedigree is shown in Figure 1, highlighting the absence of any known relatives with episodic weakness, paralysis, or documented hypokalemia. Formal segregation analysis was not possible because parental samples were not available for genetic testing. She reported occasional alcohol use but denied smoking or illicit drug use. On examination, she demonstrated minimal weakness in the lower extremities, with otherwise normal findings. Her office vital signs were within normal limits, including blood pressure 118/78 mmHg and heart rate 68 bpm. Initial laboratory workup (Table 1) revealed normal CRP, ESR, CBC, and CMP. Antinuclear antibody (ANA) was positive, renin was low, and the aldosterone‐to‐renin ratio was elevated, while serum potassium was within the normal range. The patient states that she has not made any modifications to her diet since being discharged from the ED.

**TABLE 1:** Initial laboratory results.

Hemoglobin	12.7 g/dL	Sodium	141 mmol/L
Hematocrit	39.3	Potassium	4 mmol/L
White blood cell count	5.3	Chloride	107 mmol/L
Platelet count	232	Carbon dioxide	27
MCV	83	Calcium	9.7 mg/dL
RBC	4.7	BUN	14
ESR	11	Creatinine	0.6 mg/dL
CRP	1.5	GFR	90 mL/min
ACE	18	AST	13
Glucose	101 mg/dL	ALT	14
ANA	positive	ALP	63
Plasma Renin	0.17	Aldosterone	7
Aldosterone/Renin	41.2		

## 3 Differential Diagnosis, Investigations and Treatment

The differential diagnosis included hyperaldosteronism, hypokalemia secondary to MCAS flares (via transcellular potassium shifts), and hKPP. Hyperaldosteronism was considered unlikely, as her blood pressure was normal and a 24‐h urine aldosterone was in the low‐normal range (6.7 mg/L; reference 2.1–21.3). Given her presentation and history, hKPP and MCAS flares remained a leading consideration, and she was referred for genetic testing utilizing the Renasight gene panel offered by Natera for kidney diseases [10]. Comprehensive genetic analysis did not identify any pathogenic or likely pathogenic variants associated with HPP. However, a heterozygous allelic variant was detected in the *CACNA1S* gene: *CACNA1S* (NM_000069.3:c.3844G > T; p.Ala1282Ser), which was classified by Natera as a variant of uncertain significance (VUS). The *CACNA1S* gene encodes the α1‐subunit of the L‐type voltage‐dependent calcium channel, which is implicated in hypokalemic periodic paralysis type 1, typically with autosomal inheritance (OMIM 170400). The identified missense variant (p.Ala1282Ser) is ultra‐rare (allele frequency < 0.01% in gnomAD), but its pathogenicity has not been established. It resides within transmembrane domain III of the CACNA1S protein, which contributes to essential channel properties, like voltage sensing, gating, and ion selectivity. A change of Alanine to Serine at the 1282 position is a conservative amino acid (AA) change in terms of size; however, it introduces a small polar side chain (hydroxyl group), which could potentially alter local folding, structural interaction, and phosphorylation potential. Alanine is conserved at position 1282 across various species (including human, mouse, bovine, and blue whale; UniProt Q13698, Q02789, G3MZ07, and A0A8C0CDE2 respectively). The patient was started on oral potassium supplementation, counseled on a high‐potassium diet, and trialed on spironolactone, which she did not tolerate.

## 4 Discussion

hKPP is a type of disease that falls under the category of periodic paralysis: a type of muscle disease that is a channelopathy (dysfunction of the ion channels) [1]. Our case is notable for an atypical age and sex at presentation, an overlap with MCAS, and the identification of a previously unreported CACNA1S variant currently classified as a VUS.

hKPP is the most common type of periodic paralysis with higher prevalence among Caucasians and affecting more men [1, 2]. On the contrary, acquired hKPP due to thyrotoxicosis is more common among Asians [3]. hKPP is associated with two ion channel mutations with autosomal dominant pattern of inheritance. The calcium ion channel gene CACNA1S has been associated with 60%–80% of patient cases. The second gene SCN4a, encoding for the skeletal muscle voltage‐gated sodium channel alpha subunit, associated with about 15%–20% of cases. The etiology of approximately 10%–15% cases of hKPP remains uncertain [1, 2]. CACNA1S specifically contributes to the skeletal muscle excitation‐contraction coupling in movement. Although the direct mechanism for hKPP is unknown, it is understood that the mutation dampens the rate of calcium influx, which potentially causes less sensitivity to the potassium gradient [4]. This leads to the myotonic contractions that are prominent in hKPP. Mutations in CACNA1S have unequal penetrance with a stronger penetrance in males [2, 3, 4, 5]. SCN4a mutations lead to excessive depolarization and muscle contraction [4]. Unlike CACNA1s mutations, there is equal penetrance in males and females [6]. In both cases, potassium levels drop below 3.5 meq/Liter, leading to sustained depolarization. In addition, hKPP can also be acquired. Although there are multiple causes, a principal contributor is thyrotoxicosis via stimulation of beta receptors [7]. The thyroid hormone and beta receptors work synergistically to activate the Na‐K‐ATPase channels. This can prompt a drive of potassium into the cells, leading to a deficiency of potassium in the blood [8].

Typically, hKPP presents in childhood to adolescence, mainly peaking around 15–35 years of age [1, 2]. Acquired hKPP occurs during late adolescence. These attacks are sudden in onset and are accompanied by generalized weakness [1]. Attacks can vary in duration, from a few hours to a few days, and can occur sporadically. They lessen in frequency with aging. The condition presents more insidiously in women [2]. These attacks are triggered by excitement, a high carbohydrate diet, stress, exercise, consumption of alcohol, and any medications that can decrease potassium levels such as laxatives, insulin, or steroids [1, 2]. Physical examination findings in hKPP vary depending on whether the patient is evaluated during an attack. During symptomatic episodes, patients may demonstrate hyporeflexia, fasciculations, and flaccid weakness, particularly in the lower extremities. Between attacks, the examination is often normal [2].

Although hKPP is typically diagnosed in childhood or early adolescence, our patient presented in her early 30s with generalized weakness, muscle fatigue, and muscle spasms associated with episodic hypokalemia. Her symptoms often persisted for several hours to days before resolving. Weaknesses and muscle cramps were most frequently noted after dance classes and improved with rest. Physical examination was largely unremarkable, as she was evaluated during a period of mild symptoms with normal serum potassium levels, although mild lower‐extremity stiffness and weakness were noted. Laboratory evaluation did not reveal evidence of hyperthyroidism, with TSH levels remaining within the normal range.

In typical cases, diagnosis relies mainly on clinical history, as symptoms are episodic and often accompanied by a positive family history reflecting an underlying genetic cause. When the clinical presentation is characteristic and a clear family history is present, additional diagnostic testing may not be required. Initial evaluation should include a complete metabolic panel, as hypokalemia is central to diagnosis; serum potassium levels below 3.5 mEq/L, and often below 2.5 mEq/L during acute attacks, strongly support hKPP. Measurement of TSH is also warranted due to the association between hKPP and hyperthyroidism, particularly in patients of Asian descent [8]. If a patient is not experiencing an attack, an attack could be induced by induction of insulin or corticotropin as these can precipitate lower potassium levels and symptoms [2]. If the patient is undergoing an acute attack, one could confirm diagnosis of hKPP through an EMG. EMG would reveal reduced amplitude of muscle potential in proximal muscles. An alternative diagnostic test is a long exercise test. In this test, a nerve stimulator would be attached to the abductor digiti minimi [9]. Action potentials are measured during physical exercise. According to Riberio et al., this test shares a specificity of 71%–95%, with higher sensitivity correlating to patients presenting with more frequent attacks [9]. The most specific test would be genetic testing for mutations of the CACNA1 or the SCN4 channels, which would assist greatly in diagnosis; however, testing is often not timely and may be too expensive [1]. Table 2 summarizes the age, gender, initial symptoms, potassium levels, and methods used to confirm the diagnosis in previously reported cases of hKPP.

**TABLE 2:** Previous case reports reporting hypokalemic periodic paralysi*s*.

Author	Case	Symptoms	Potassium	Diagnosis confirmation
Noor et al. [11]	15 y/o Male	Acute flaccid paralysis post‐exercise; proximal and distal muscle weakness	2.2 mmol/L	Clinical improvement with potassium supplementation; likely HPP
Kumarajothy et al. [12]	21 y/o Female	Sudden generalized weakness, history of low potassium since age 15	2.1 mmol/L	Clinical diagnosis; genetic testing mentioned but not done
Soule et al. [13]	29 y/o Male	Sudden paralysis with proximal weakness and tachycardia	1.6 mmol/L	Not confirmed; no genetic testing
Jaishi et al. [14]	25 y/o Male	Acute limb weakness post‐heavy meal; similar past episode	Not reported (treated before)	Nerve conduction normal; confirmed by technetium scan
Zhang et al. [15]	29 y/o Male	Recurrent AM weakness, chest tightness, dyspnea	1.6 mmol/L (at attack)	Confirmed SCN4A mutation, positibe for Graves disease
Chen et al. [16]	32 & 38 y/o Males	Generalized weakness post‐injection (B vitamins, steroids)	1.6 and 1.8 mmol/L	Met diagnostic criteria; no genetic testing
Tai et al. [17]	26 y/o Male	B/L lower limb weakness post‐dexamethasone injection	2.0 mmol/L	No genetic testing; diagnosis based on clinical presentation
Castañeda et al. [18]	9 y/o Male	Flaccid paralysis triggered by carbohydrates; worsened with acetazolamide	2.4 mmol/L	Novel ATP1A2 gene mutation found

**FIGURE 2:**
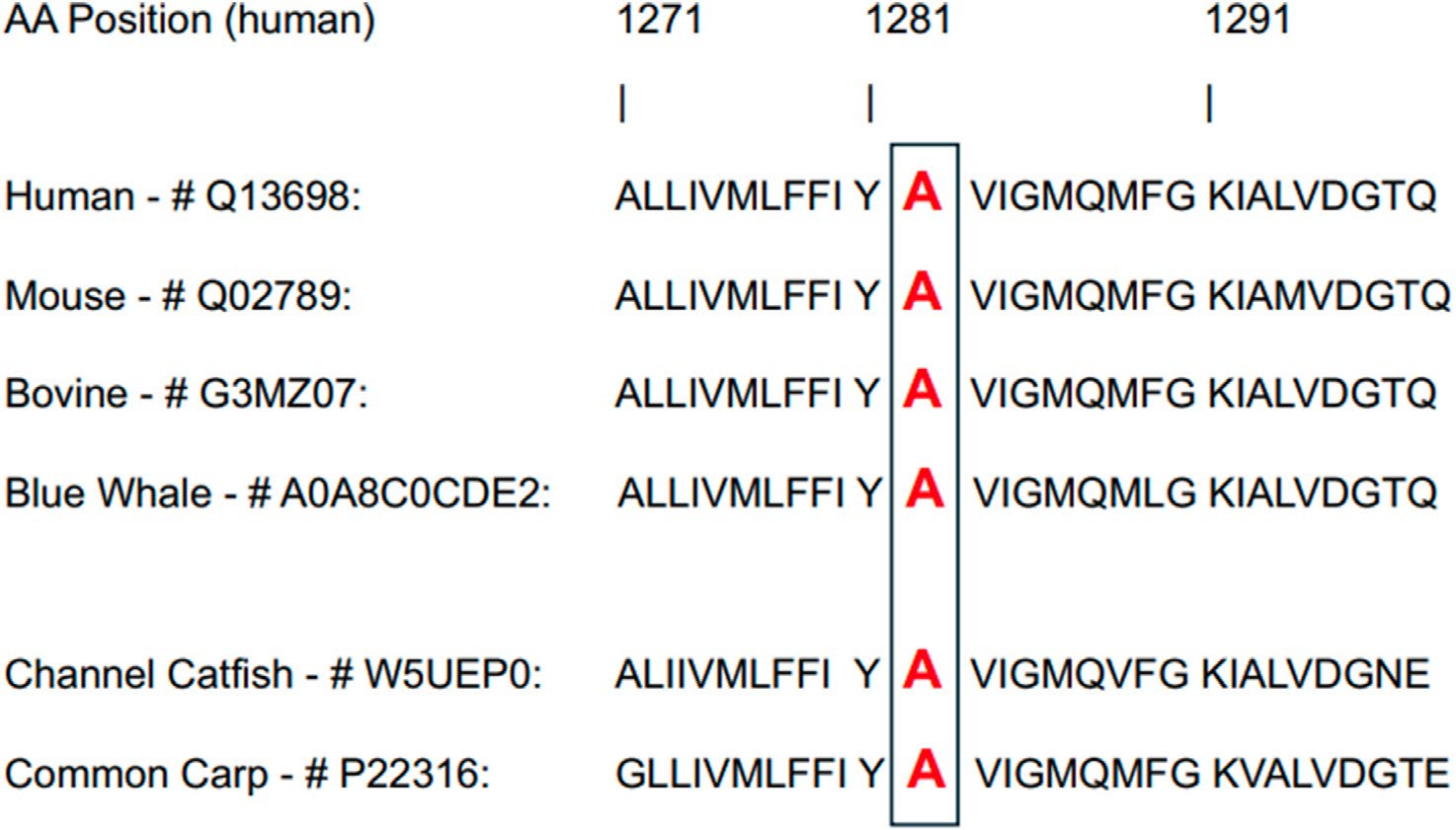
Multiple sequence alignment of CACNA1S orthologs in mammals (human, mouse, bovine, blue whale), and two teleost fish (channel catfish, common carp), with Uniprot #. Alanine (A) at Amino Acid (AA) position corresponding to human residue 1282 (boxed) in conserved in all six species. The p.Ala1282Ser variant alters a highly conserved residue within conserved transmembrane domain III, supporting possible functional relevance.

Our patient denied any family history of hPKK; however, the nature and duration of her symptoms, along with episodic hypokalemia precipitated by physical activity, were consistent with hKPP. Given the overlap of her symptoms with mast cell activation syndrome (MCAS), genetic testing was pursued as a more specific and feasible diagnostic tool. Interestingly, she was found to have a heterozygous CACNA1S variant (NM_000069.3:c.3844G > T; p.Ala1282Ser). Notably, this specific mutation has not been previously reported and was classified as a VUS (Dahl et al., JASN, December 1, 2023, PMID: 37794654); regardless, the patient's clinical presentation are more consistent with hKPP than with MCAS. Variant classification was performed according to the 2015 ACMG/AMP guidelines, incorporating population, computational, and functional data where available. The p.Ala1282Ser variant is extremely rare in gnomAD (allele frequency < 0.01%) and affects a highly conserved residue in transmembrane domain III, which is known to harbor pathogenic CACNA1S variants associated with HypoPP. However, there are no published functional studies or segregation data for this specific variant. In ClinVar, p.Ala1282Ser is currently reported with conflicting interpretations of pathogenicity, and an automated ACMG re‐evaluation based on available data predicts a “likely benign” classification. In our report, we therefore conservatively retain the designation “variant of uncertain significance” while noting that the patient's phenotype is compatible with CACNA1S‐related hypokalemic periodic paralysis. To assess evolutionary conservation, we performed a multiple sequence alignment of CACNA1S orthologs including mammals (human, mouse, bovine, whale) and bony fish (teleosts), which demonstrated that alanine at position 1282 is strictly conserved in transmembrane domain III across all examined species (Figure 2). This supports the potential functional importance of this residue.

In silico structural modeling and more advanced computational analyses might further clarify the potential impact of the p.Ala1282Ser substitution on channel structure and function, but these were beyond the scope and resources of this single‐case report. Likewise, segregation studies (including parental testing) were not feasible, which limits our ability to formally establish or refute causality for this variant.

The case presented here was previously diagnosed with MCAS by her allergist due to episodes of rashes and allergic symptoms. MCAS can sometimes present with features resembling those of hKPP. According to a study by Afrin et al., patients with MCAS commonly experience fatigue (83%) and presyncope (71%), both of which were also reported by our patient. Additionally, the study found that 41% of patients demonstrated hypokalemia during MCAS episodes, likely due to transcellular potassium shifts. Patients can also exhibit eosinophilia during acute attacks; however, our patient's laboratory results during her clinic visits showed normal eosinophil levels. MCAS shares several triggers with hKPP, including emotional stress, heat, exercise, and high‐carbohydrate foods [19, 20]. Although fatigue, weakness, and presyncope have been reported during MCAS flares, these episodes are typically associated with urticaria, angioedema, and skin flushing, as well as gastrointestinal symptoms such as diarrhea, nausea, and vomiting, none of which were observed in our patient. We did not measure serum tryptase levels, which can be elevated in patients with MCAS during an acute attack [19]. Given that our patient has a confirmed diagnosis of MCAS and overlapping clinical and laboratory features of both MCAS and hKPP, it remains unclear whether her symptoms are primarily due to MCAS or hKPP. However, the presence of a heterozygous CACNA1S variant, documented hypokalemia, and the absence of typical MCAS manifestations such as flushing, urticaria, or other allergic symptoms suggest that hKPP is the more likely diagnosis. Table 3 summarizes and compares the etiology, pathophysiology, presentation, diagnosis, and management of hKPP and MCAS.

**TABLE 3:** Comparison of hypokalemic periodic paralysis and mast cell activation syndrome.

	hPKK	MCAS
Etiology	Hereditary causes: mutation in sodium or calcium channels in skeletal muscle. Acquired causes: thyrotoxicosis, steroid use [21, 22].	Alcohol, heat, medications (antibiotics, NSAIDS, morphine, anesthesia), bug stings, exercise, and stress. Can be idiopathic or due to a mutation in c‐KIT gene [23, 24].
Pathophysiology	Mutations in CACNA1S (70%) or SCN4A (30%) genes disrupt the electrical current of the skeletal muscle voltage channels leading to no muscle stimulation and subsequent muscle flaccidity [21, 22]	The mast cells are activated inappropriately. This can come from a mutation in c‐KIT, controlling proliferation of mast cells. The pathophysiology is not solidified [24].
Presentation	Focal or generalized episodes of paralysis associated with generalized weakness. Hypokalemia K < 2.5 usually associated with increased insulin or epinephrine release. Attacks typically occur with high carbohydrate meals, alcohol consumption or strenuous exercise [1]	Symptoms resemble anaphylaxis, flushing of skin, nausea, vomiting, abdominal pain, angioedema, throat and chest tightness, shortness of breath, wheezing, hypotension and tachycardia [24].
Diagnosis	Genetic testing for CACNA1s or SCN4a. Measuring potassium levels during acute episode, can also measure TSH, T3, T4 to rule out thyrotoxicosis cause of hKPP Long exercise test: Induces a muscle attack after 2–5 min of exercise. EMG will measure levels of cAMP in muscle fibers (looking for at least 40% decrease in cAMP in muscle fibers) [1, 22] Induce an episode by injecting glucose or insulin [1]	Diagnosing MCAS requires 3 criteria: (1) Episodic, objective signs and symptoms consistent with MC activation in at least 2 organ systems: GI, skin, respiratory, or cardiovascular; (2) Evidence of systemic mast cell mediator release (serum tryptase increase from baselin or increased urine mast cell mediators); (3) Response to medications stabilizing mast cells Laboratory: Tryptase, KIT D816V, skin and GI biopsy, urine mediators, allergens [24, 25]
Management	Acute onset: KCl 0.5–1 mEq/kg orally. If patient needs more than 100 mEq of potassium, hospitalization with close potassium monitoring and EKG is recommended Chronic: Dichlorphenamide, acetazolamide, potassium supplementation, education about diet and exercise modification to prevent attacks [21]	Due to genetic roots, MCAS is considered incurable, so treatment is with symptom management including antihistamines, cromolyn, vitamin C, and treating underlying symptoms, or omalizumab [24, 25]

Treatment is focused on replenishing potassium stores and preventative treatment of future attacks induced by hKPP. The mainstay of treatment is oral potassium chloride supplementation [2]. Once administered, levels should be monitored for 24 h to manage response and prevent hyperkalemia [2]. If the attack is mild, a low degree of exercise can stop the muscle weakness [1]. If hKPP is acquired secondary to thyrotoxicosis, management of hyperthyroidism should take precedence. Preventative treatment can be utilized to manage hKPP. There are two options of treatment. The first line treatment is carbonic anhydrase inhibitors [1, 2]. Acetazolamide, dosed at 250 mg twice daily, can prevent future attacks. Other options are dichlorphenamide (recommended dose 50 mg twice daily) and potassium‐sparing diuretics. Recommendations for Hypokalemic Periodic Paralysis should be guided by disease severity and attack frequency. Management ranges from avoidance of trigger factors in mild cases to oral potassium supplementation in patients with frequent episodes, with close monitoring of serum potassium levels. Pharmacologic therapy may be considered in selected cases [26].

Hypokalemic periodic paralysis (hKPP) is a rare disorder that can present with nonspecific symptoms. The presence of hypokalemia, a positive family history, and identifiable triggers can provide important diagnostic clues. It is always essential to rule out secondary causes, including hyperthyroidism. Although clinical findings can be sufficient for diagnosis, genetic testing can be a valuable tool in patients without family history and other medical conditions that may present with similar features such as mast cell activation syndrome (MCAS).

## Author Contributions

**Ali Moradi:** conceptualization, writing – original draft, writing – review and editing. **Yasmin Aboutaleb:** methodology, writing – original draft, writing – review and editing. **Saba Noreen:** methodology, writing – original draft, writing – review and editing. **Abdullah Sahyouni:** methodology, writing – original draft, writing – review and editing. **Hasin Sharma:** writing – original draft, writing – review and editing. **Olugbenga Oyesanmi:** conceptualization, supervision, writing – original draft, writing – review and editing. **Ian M. Kahane:** conceptualization, supervision, writing – review and editing. **Yizhi Lin:** conceptualization, supervision, writing – original draft. **Hakan R. Toka:** conceptualization, supervision, writing – review and editing.

## Funding

The authors have nothing to report.

## Data Availability

The data supporting the findings of this study are not publicly available due to patient confidentiality and privacy concerns.

## References

[1] J. M. Statland , B. Fontaine , M. G. Hanna , et al., “Review of the Diagnosis and Treatment of Periodic Paralysis,” Muscle & Nerve 57, no. 4 (2018): 522–530. 29125635 10.1002/mus.26009 PMC5867231

[2] P. Phuyal , B. S. Bhutta , and S. Nagalli , “Hypokalemic Periodic Paralysis,” in StatPearls (StatPearls Publishing Copyright 2025, StatPearls Publishing LLC, 2025).

[3] S. Patra , P. P. Chakraborty , S. N. Biswas , and H. Barman , “Etiological Search and Epidemiological Profile in Patients Presenting With Hypokalemic Paresis: An Observational Study,” Indian Journal of Endocrinology and Metabolism 22, no. 3 (2018): 397–404. 30090734 10.4103/ijem.IJEM_633_17 PMC6063177

[4] O. Szymanowicz , A. Drużdż , B. Słowikowski , et al., “A Review of the CACNA Gene Family: Its Role in Neurological Disorders,” Diseases 12, no. 5 (2024): 90. 38785745 10.3390/diseases12050090 PMC11119137

[5] X. Y. Wang , B. W. Ren , Z. H. Yong , H. Y. Xu , Q. X. Fu , and H. B. Yao , “Mutation Analysis of CACNA1S and SCN4A in Patients With Hypokalemic Periodic Paralysis,” Molecular Medicine Reports 12, no. 4 (2015): 6267–6274. 26252573 10.3892/mmr.2015.4201

[6] D. Sternberg , T. Maisonobe , K. Jurkat‐Rott , et al., “Hypokalaemic Periodic Paralysis Type 2 Caused by Mutations at Codon 672 in the Muscle Sodium Channel Gene SCN4A,” Brain 124, no. Pt 6 (2001): 1091–1099. 11353725 10.1093/brain/124.6.1091

[7] E. P. Rhee , J. A. Scott , and A. S. Dighe , “Case Records of the Massachusetts General Hospital. Case 4‐2012. A 37‐Year‐Old Man With Muscle Pain, Weakness, and Weight Loss,” New England Journal of Medicine 366, no. 6 (2012): 553–560. 22316449 10.1056/NEJMcpc1110051

[8] Z. A. Hannouneh , C. E. Cervantes , C. J. Sperati , and M. Hanouneh , “Familial Hypokalemic Periodic Paralysis: A Case Induced by Concurrent Hyperthyroidism,” BMC Nephrology 25, no. 1 (2024): 315. 39333966 10.1186/s12882-024-03749-x PMC11429431

[9] A. Ribeiro , K. J. Suetterlin , I. Skorupinska , et al., “The Long Exercise Test as a Functional Marker of Periodic Paralysis,” Muscle & Nerve 65, no. 5 (2022): 581–585. 34817893 10.1002/mus.27465 PMC7614949

[10] N. K. Dahl , M. S. Bloom , F. T. Chebib , et al., “The Clinical Utility of Genetic Testing in the Diagnosis and Management of Adults With Chronic Kidney Disease,” Journal of the American Society of Nephrology 34, no. 12 (2023): 2039–2050. 37794564 10.1681/ASN.0000000000000249 PMC10703084

[11] S. Noor , A. J. Rasooly , S. M. Alikozai , et al., “Hypokalemic Periodic Paralysis in a Teenage Boy After an Intense Period of Exercise: A Rare Case Report,” Clinical Case Reports 11, no. 11 (2023): e8201. 38028058 10.1002/ccr3.8201 PMC10645604

[12] R. Kumarajothy , A. Ul‐Haq , and M. M. Akhtar , “A Case Report on Hypokalemic Periodic Paralysis,” Cureus 16, no. 10 (2024): e72652. 39478764 10.7759/cureus.72652 PMC11522841

[13] B. R. Soule and N. L. Simone , “Hypokalemic Periodic Paralysis: A Case Report and Review of the Literature,” Cases Journal 1, no. 1 (2008): 256. 18939979 10.1186/1757-1626-1-256 PMC2584072

[14] P. PoudelJaishi , S. K. Neupane , and P. K. Neupane , “Case Report: Hyperthyroid Hypokalemic Periodic Paralysis,” Annals of Medicine and Surgery 78 (2022): 103759. 35620041 10.1016/j.amsu.2022.103759 PMC9127175

[15] Z. Zhang and B. Xiao , “Case Report: SCN4A p.R1135H Gene Variant in Combination With Thyrotoxicosis Causing Hypokalemic Periodic Paralysis,” Frontiers in Neurology 13 (2022): 1078784. 36733446 10.3389/fneur.2022.1078784 PMC9886676

[16] B. Chen , C. J. Counts , P. Maresca , H. A. Greller , and M. J. Heller , “Simultaneous Cases of Familial Hypokalemic Periodic Paralysis Induced by Illicit Injection of Betamethasone,” Journal of Emergency Medicine 70 (2025): 92–97. 39952820 10.1016/j.jemermed.2024.09.021

[17] H. T. Tai , P. T. Lee , and S. H. Ou , “Steroid‐Induced Hypokalemic Periodic Paralysis: A Case Report and Literature Review,” BMC Nephrology 24, no. 1 (2023): 70. 36964512 10.1186/s12882-023-03131-3 PMC10039554

[18] M. Sampedro Castañeda , E. Zanoteli , R. S. Scalco , et al., “A Novel ATP1A2 Mutation in a Patient With Hypokalaemic Periodic Paralysis and CNS Symptoms,” Brain 141, no. 12 (2018): 3308–3318. 30423015 10.1093/brain/awy283 PMC6262219

[19] Ö. Özdemir , G. Kasımoğlu , A. Bak , H. Sütlüoğlu , and S. Savaşan , “Mast Cell Activation Syndrome: An Up‐To‐Date Review of Literature,” World Journal of Clinical Pediatrics 13, no. 2 (2024): 92813. 38948000 10.5409/wjcp.v13.i2.92813 PMC11212760

[20] L. B. Afrin , S. Self , J. Menk , and J. Lazarchick , “Characterization of Mast Cell Activation Syndrome,” American Journal of the Medical Sciences 353, no. 3 (2017): 207–215. 28262205 10.1016/j.amjms.2016.12.013 PMC5341697

[21] K. Gadalla and C. Anastasopoulou , “Hypokalemic Periodic Paralysis,” in StatPearls (StatPearls Publishing Copyright 2025, StatPearls Publishing LLC, 2025). 32644604

[22] S. Paydas , M. A. Gergerli , and A. Celik , “Hypokalemic Periodic Paralysis, a Rare Yet Critical Condition: A Case Report,” Medicine International 5, no. 2 (2025): 21. 40013235 10.3892/mi.2025.220 PMC11863296

[23] P. Bonadonna , M. Pagani , W. Aberer , et al., “Drug Hypersensitivity in Clonal Mast Cell Disorders: ENDA/EAACI Position Paper,” Allergy 70, no. 7 (2015): 755–763. 25824492 10.1111/all.12617

[24] M. Castells , M. P. Giannetti , M. J. Hamilton , et al., “Mast Cell Activation Syndrome: Current Understanding and Research Needs,” Journal of Allergy and Clinical Immunology 154, no. 2 (2024): 255–263. 38851398 10.1016/j.jaci.2024.05.025 PMC11881543

[25] G. J. Molderings , B. Haenisch , S. Brettner , et al., “Pharmacological Treatment Options for Mast Cell Activation Disease,” Naunyn‐Schmiedeberg's Archives of Pharmacology 389, no. 7 (2016): 671–694. 27132234 10.1007/s00210-016-1247-1 PMC4903110

[26] S. L. Venance , S. C. Cannon , D. Fialho , et al., “The Primary Periodic Paralyses: Diagnosis, Pathogenesis and Treatment,” Brain 129, no. Pt 1 (2006): 8–17. 16195244 10.1093/brain/awh639

